# Drug-Induced Thrombotic Microangiopathy due to Cumulative Toxicity of Ixazomib

**DOI:** 10.1155/2018/7063145

**Published:** 2018-07-03

**Authors:** Suheil Albert Atallah-Yunes, Myat Han Soe

**Affiliations:** Department of Medicine, Baystate–University of Massachusetts Medical School, Springfield, MA, USA

## Abstract

Drug-induced thrombotic microangiopathies (DTMAs) are increasingly being recognized as an important category of thrombotic microangiopathies (TMAs). Cancer therapeutic agents including proteasome inhibitors (PIs) are among the most common medications reported to cause DTMA. PIs could cause DTMA either by an immune mechanism or dose-dependent/cumulative toxicity. Eleven cases of DTMA have been reported with bortezomib and carfilzomib. To the best of our knowledge, only one case of DTMA has been reported with ixazomib due to an immune-mediated mechanism. Here, we report the first case of ixazomib-induced DTMA due to cumulative toxicity rather than immune-mediated mechanism. In this article, we discuss the precipitating factors for cumulative toxicity of ixazomib, resulting in DTMA, diagnostic workup, and management of DTMA. We also discuss clinical reasoning based analysis of DTMA versus cancer-associated TMA as well as DTMA versus cyclic thrombocytopenia seen in PI use.

## 1. Introduction

Thrombotic microangiopathies are a group of disorders characterized by thrombocytopenia, microangiopathic hemolytic anemia, and ischemic end organ damage mostly involving the kidneys and brain caused by disseminated occlusive microvascular thrombosis [1]. TMA is well known to occur in the setting of thrombotic thrombocytopenic purpura (TTP) and hemolytic uremic syndrome (HUS). Other causes of TMA include atypical HUS, various malignancies, rheumatological diseases, and medications [1, 2]. TMA caused by malignancy has been mostly reported with adenocarcinomas metastasizing to bone marrow. Common solid tumors that have been linked to cancer-induced TMA include gastric, breast, lung, and prostate adenocarcinomas, with gastric adenocarcinoma being the most reported. It has also been reported with hematologic malignancies such as lymphoma and multiple myeloma [3]. TMA caused by drugs is called drug-induced TMA (DTMA) [4], and cancer therapeutic agents are among the most common medications reported to cause DTMA. Many cases of DTMA linked to bortezomib and carfilzomib have been reported [5].  Table 1 illustrates the most common cancer therapeutic agents known to cause DTMA, with the most common mechanism being either toxic or immune mediated or both [6–8].

Proteasomes are multicatalytic enzymatic complexes located in both nucleus and cytoplasm that aid in intracellular protein homeostasis by degradation and recycling of proteins [9]. They are an important target in the treatment of multiple myeloma as plasma cells producing paraproteins are highly dependent on these enzyme complexes for survival. PIs prevent degradation of proapoptotic factors and permit activation of programmed cell death in myeloma cells [10]. They also act by stabilization of nuclear factor kB (NF-kB) which ultimately decreases the proliferation of myeloma cells. Bortezomib and carfilzomib are the first and second generation PIs approved by FDA for treatment of multiple myeloma [11–14]. Side effects with these two PIs include gastrointestinal disturbances, peripheral neuropathy, and cyclic thrombocytopenia. The presence of a wide range of side effects and the emergence of chemoresistance required the introduction of newer generation of PIs with fewer side effects such as ixazomib.

Ixazomib is the third generation proteasome inhibitor used in the treatment of refractory or relapsing multiple myeloma in combination with dexamethasone and lenalidomide. It has less side effects and several advantages over bortezomib and carfilzomib [15]. Studies show that ixazomib is associated with less frequent and less severe peripheral neuropathy [16, 17]. Another advantage is that ixazomib could be administered orally [18]. The most common side effects reported with ixazomib are gastrointestinal disturbances including nausea, vomiting, diarrhea, and constipation [15].

Thrombocytopenia is also another commonly reported adverse effect as PIs interfere directly with the budding of megakaryocytes rather than causing direct damage to the bone marrow [19]. Thrombocytopenia commonly seen with PIs is called cyclic thrombocytopenia in which the platelet count usually nadirs around day 11 of the cycle and then improves. The decrease in the platelet count is rarely severe, and the platelet count usually nadirs to a minimum of 60% of baseline [19]. The literature also provides a strong evidence of PIs causing TMA in which thrombocytopenia is more severe and accompanied by intravascular hemolytic anemia and renal dysfunction. Clinicians should be familiar with this thrombocytopenia pattern and severity in order to differentiate between benign cyclic thrombocytopenia and TMA in the setting of PI use. When proven to be TMA, another challenge is to differentiate whether TMA is caused by the PI or the multiple myeloma itself as both are managed differently. It is crucial to be familiar with this very rare side effect of ixazomib and other PIs causing TMA, as the principle treatment is to stop the implicated medication [2]. To the best of our knowledge, only one case of immune-mediated DTMA caused by ixazomib has been reported [6]. Here, we report the first case of ixazomib-induced DTMA due to cumulative toxicity rather than immune-mediated mechanism.

## 2. Case Description

**Figure 1:**
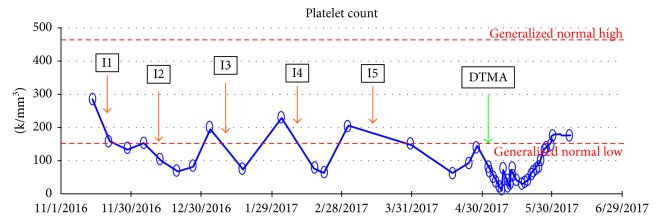
Cyclic thrombocytopenia in relation to cycles of ixazomib indicated by orange arrows (I1–I5) and the onset of DTMA indicated by green arrow.

A 71-year-old female with multiple myeloma status after 5 cycles of ixazomib, lenalidomide, and dexamethasone, chronic kidney disease stage III, previous stroke, hypertension, gout, and peripheral arterial disease presented to the hospital with generalized weakness, vomiting, and diarrhea as well as acute on chronic kidney injury in which serum creatinine and creatinine clearance (CrCl) were 3.3 mg/dl and 15 ml/min, respectively (baseline creatinine of 1.9 mg/dl with CrCl of 30 ml/min). Blood test showed thrombocytopenia with a platelet count of 84000/dl and anemia with hemoglobin of 12 g/dl. Regarding multiple myeloma, she was diagnosed with kappa light chain multiple myeloma with extensive lytic lesions in bones as well as renal dysfunction a few years ago. Diagnosis was made by bone marrow biopsy which demonstrated 80%–90% cellular marrow with 61% plasma cells. FISH study was abnormal for chromosome 1q, chromosome 13q, and 17p deletion. Based on patient's average CrCl of 30 ml/min, ixazomib was started at a dose of 3 mg on days 1, 8, and 15 of a 28-day treatment cycle along with lenalidomide and dexamethasone. After the second cycle of ixazomib, the patient had been having intermittent GI disturbances including diarrhea, and biweekly blood test revealed thrombocytopenia with a nadir of about 75000/dl (Figure 1), which were both attributed to ixazomib. Ixazomib was held on admission due to significant vomiting, abdominal pain, and diarrhea. *Clostridium difficile* toxin and stool culture were negative, ruling out infectious causes. One week after admission, the platelet count decreased dramatically to 9000/dl from 84000/dl on admission. The patient also developed intravascular hemolysis evident by an elevated LDH level (1366 units/L), decreased haptoglobin level (10 mg/dl), elevated total bilirubin (1.6 mg/dl), and indirect bilirubin (1.3 mg/dl). Peripheral blood smear also showed profound schistocytes. Coomb's test was negative, and DIC was ruled out as the fibrinogen level was normal (521 mg/dl). Acute thrombocytopenia, Coomb's negative hemolytic anemia with profound schistocytes, and acute renal injury raised the concern for TMA. Given the high morbidity of TMA, the patient received fresh frozen plasma and underwent plasmapheresis while further workup was in progress. Normal ADAMTS13 activity ruled out TTP. Normal complement levels and negative stool culture made atypical HUS and HUS less likely. Plasmapheresis was stopped after 5 days due to lack of clinical improvement and negative workup for TTP. Approximately three weeks after the onset of TMA, the platelet count started to improve spontaneously with supportive management. The gradual and spontaneous improvement in the platelet count pointed suspicion away from malignancy-induced TMA and favored DTMA caused by cumulative toxicity of ixazomib, likely precipitated by acute renal dysfunction and hypoproteinemia from malnutrition and chronic diarrhea related to ixazomib side effect. The presentation of this patient was consistent with ixazomib-induced DTMA from cumulative toxicity as the clinical picture of TMA improved after stopping ixazomib, independently of plasmapheresis. Also, the lack of recurrence of TMA after stopping ixazomib supported the diagnosis in our case.

## 3. Discussion

In the presence of possible offending medication, DTMA should be suspected in patients having acute onset thrombocytopenia, nonimmune intravascular hemolytic anemia with schistocytes and renal injury, with resolution of TMA after stopping the medication and ruling out other causes of TMA. Diagnosis of DTMA is supported if TMA reoccurs after reintroducing the drug. There is no specific time frame in which DTMA develops after introducing the medication. It could range from days to years after the initial dose [5].

The literature describes two main mechanisms causing DTMA which are immune-mediated and dose-dependent toxicity [5, 6, 20]. Immune-mediated reactions are also called idiosyncratic reactions as it involves the formation of reactive antibodies against drugs that cause damage to the endothelium leading to TMA [20]. DTMA due to an idiosyncratic reaction has been mostly reported with quinidine [21] and quetiapine [22]. However, DTMA occurring due to a toxic reaction is usually a dose-dependent toxicity, and results from either direct toxicity of the drug to microvasculature or inhibition of VEGF leading to endothelial damage [6, 20]. Most case reports linking DTMA to PIs favor immune-mediated mechanism as the cause of DTMA (Table 1) although drug-dependent antibodies were not documented.

Eleven cases of DTMA have been reported with bortezomib and carfilzomib [5]. To the best of our knowledge, it has been reported only once with ixazomib due to immune- mediated mechanism [6]. In this case report, we report the second case of DTMA caused by ixazomib. Contrary to the first case report, ixazomib-induced DTMA in our case is likely due to cumulative toxicity. Lack of improvement with plasmapheresis in our case makes immune-mediated mechanism less likely. According to pharmacokinetic data, 99% of ixazomib is plasma protein bound, and the mean area under the curve (AUC) could be 39% higher in patients with severe renal impairment (creatinine clearance <30 ml/min) [18]. We assume that severe renal impairment and hypoproteinemia (total protein: 4.9 g/dl; albumin: 2.4 g/dl) from persistent diarrhea and malnutrition might have precipitated the cumulative toxicity of ixazomib in our case.

TMA could be also caused by malignancies including multiple myeloma [23, 24]. It is important to differentiate between cancer-related TMA and DTMA in cancer patients receiving chemotherapeutic agents such as in multiple myeloma patients receiving PIs. TMA caused by multiple myeloma is usually treated with chemotherapy including PIs. Previous case reports suggest that TMA caused by monoclonal gammopathy resolved with bortezomib. This contrasts with cases of DTMA caused by bortezomib in which TMA resolved after stopping the medication. In our case, paraproteins were close to the normal limit at the time of TMA occurrence. Hemolytic anemia and thrombocytopenia resolved after stopping ixazomib and never reoccurred after that, suggesting TMA was caused by ixazomib rather than multiple myeloma itself.

One of the most commonly reported side effects of ixazomib is thrombocytopenia [15]. It is also crucial to differentiate between cyclic thrombocytopenia and thrombocytopenia due to DTMA. In general, cyclic thrombocytopenia related to PIs is caused by a direct effect on megakaryocyte function and the platelet budding from bone marrow rather than direct damage to the bone marrow with a nadir platelet count at day 11 of the cycle and resolving just before the next cycle [19], as seen in this case (Figure 1). This coincides with the elimination half-life of ixazomib of 7.5 days [15]. Cyclic thrombocytopenia is rarely severe and usually does not require platelet transfusion. This contrasts to the dramatic thrombocytopenia seen in DTMA. In our case, the patient developed cyclic thrombocytopenia after introducing ixazomib with a nadir platelet count of 75000/dl which usually improved before the next cycle of chemotherapy. The dramatic decrease in the platelet count during this hospitalization raised the concern for DTMA especially in the presence of conclusive hemolysis workup with profound schistocytes and acute renal dysfunction.

Pathophysiology behind which PIs cause DTMA is still unclear. The most reported theory in the literature suggests that PIs stabilize and inhibit NF-kB which decreases the production of VEGF leading to microvascular injury. This theory supports cumulative dose-dependent toxicity of PIs. Other theories suggest that PIs act as proinflammatory agents leading to production of TNF and IL-6 which induce the production of autoantibodies against ADAMTS13 [6, 25]. However, the latter theory is unlikely to be the cause of DTMA in our case as our patient had normal levels of ADAMTS13, and also platelet count did not improve with plasmapheresis. Current data report more cases of DTMA with carfilzomib than bortezomib, as carfilzomib irreversibly inhibits proteasomes while bortezomib causes reversible inhibition of proteasomes [20]. The only treatment of DTMA is immediate withdrawal of the implicated agent [2]. In our case, thrombocytopenia did not improve despite 5 days of plasmapheresis. It improved independently about 20 days after stopping ixazomib, which reflects approximately three elimination half-lives of ixazomib. Outpatient records showed that the patient never developed TMA after stopping ixazomib.

The question of whether to reintroduce PIs after causing DTMA depends on the mechanism of DTMA. If DTMA is caused by immune-mediated mechanism, the implicated PI should never be reintroduced again, even with lower doses. However, if DTMA is mediated by dose-dependent cumulative toxicity as in our case, it could be reintroduced with caution. The decision must balance the potential risk of recurrent DTMA and the potential benefits of restarting the medication [26]. Based on our experience from this case, medication dosage, renal function, and total protein/albumin levels should also be taken into consideration in the decision-making process. After the resolution of DTMA, our patient has regular follow-up visits every two months for a year till now. Her blood counts have been stable and paraproteins have not been elevated, indicating nonprogressive disease. Hence, ixazomib is not reintroduced again.

## 4. Conclusion

DTMA is a very rare side effect of PIs. Ixazomib is indicated in the treatment of refractory and relapsing multiple myeloma. DTMA due to ixazomib should be suspected in the setting of TMA occurring after introducing ixazomib with no evidence of other causes of TMA. The literature reported only one case of ixazomib-induced DTMA due to immune-mediated mechanism, whereas this case highlights the cumulative toxicity of ixazomib causing DTMA. Clinicians should pay attention to changes in renal function, nutrition status, and total protein/albumin level in addition to side effect profile in the management of patients being treated with PIs to prevent cumulative toxicity which could lead to DTMA. It is also important to differentiate DTMA from malignancy-induced TMA especially in multiple myeloma patients treated with PIs such as ixazomib, as both are treated differently.

## Figures and Tables

**Table 1:** Common anticancer chemotherapeutic agents causing drug-induced thrombotic microangiopathy via immune-mediated mechanism or dose-dependent toxicity or both [4, 7].

Cancer therapeutic agent	Immune-mediated mechanism	Dose-dependent toxicity
Bevacizumab	—	X
Bortezomib	X	—
Carfilzomib	X	—
Ixazomib	X	—
Gemcitabine	X	X
Mitomycin	—	X
Oxaliplatin	X	—
Pentostatin	—	X
Sunitinib	—	X
Imatinib	X	—
Docetaxel	—	X
